# Application of artificial neural network and support vector regression in predicting mass of ber fruits (*Ziziphus mauritiana* Lamk.) based on fruit axial dimensions

**DOI:** 10.1371/journal.pone.0245228

**Published:** 2021-01-07

**Authors:** Mahmoud Abdel-Sattar, Abdulwahed M. Aboukarima, Bandar M. Alnahdi

**Affiliations:** 1 Department of Plant Production, College of Food and Agriculture Sciences, King Saud University, Riyadh, Saudi Arabia; 2 Pomology Department, Faculty of Agriculture, Alexandria University, Alexandria, Egypt; 3 Department of Agricultural Engineering, College of Food and Agriculture Sciences, King Saud University, Riyadh, Saudi Arabia; 4 Agricultural Engineering Research Institute, Agricultural Research Centre, Giza, Egypt; Vietnam National University, VIET NAM

## Abstract

Fruit quality attributes are important factors for designing a market for agricultural goods and commodities. Support vector regression (SVR), MLR, and ANN models were established to predict the mass of ber fruits (Ziziphus mauritiana Lamk.) based on the axial dimensions of the fruit from manual measurements of fruit length, minor fruit diameter, and maximum fruit diameter of four ber cultivars. The precision and accuracy of the established models were assessed given their predicted values. The results revealed that using the validation dataset, the developed ANN (R^2^ = 0.9771; root mean square error [RMSE] = 1.8479 g) and SVR (R^2^ = 0.9947; RMSE = 1.8814 g) models produced better results when predicting ber fruit mass than those obtained by the MLR model (R^2^ = 0.4614; RMSE = 11.3742 g). In estimating ber fruit mass, the established SVR and ANN models produced more precise prediction values than those produced by the MLR model; however, the performance differences between the SVR and ANN models were not clear.

## Introduction

*Ziziphus mauritiana* Lamk. originates from the *Rhamnaceae* family and is also known as Indian jujube. The ber tree can be grown in tropical and sub-tropical areas [1, 2] and can survive critical conditions, such as water-logging, drought, and salinity [3]. Ber requires minimum agricultural inputs to grow [4]. This fruit has variable sizes and shapes depending on the cultivars. Ber fruits are a popular fruit in some parts of Saudi Arabia, and are appreciated by consumers due to their quality. They are rich in a variety of vitamins and minerals [5, 6], and their nutritional value is high. In addition, their prices are higher than those of other fruits.

Physical attributes of agricultural products are important for the design of grading and sorting equipment, handling materials in different mechanical systems, and various packaging and processing machinery [7, 8]. Khojastehnazhand et al. [9] studied various qualities of fresh produce, as these attributes are often used to estimate water loss, quantity of pesticide applications, heat transfer, ripeness index to predict the optimal harvest time, respiration rates, evaluation of fruit growth and quality, grading, and so on. Thus, numerous models have been prepared to predict physical attributes from the measurement of other attributes. Models for predicting fruit mass based on the measurement of physical features have been previously published [10–20]. All of the available models were developed based on a combination of different physical attributes in different forms using statistical approaches, such as regression analysis. Thus, in data modeling in fruit grading systems, the artificial intelligence technique must be mended as different variables are fed as inputs in such systems and accuracy and precision are required. In the fruit mass modeling literature, there are several research papers on the use of artificial neural network (ANN) and multiple linear regression (MLR) approaches for predicting fruit mass, while the use of support vector machine (SVM) method as a new alternative approach to MLR and ANN models is still not fully investigated in ber fruits (Ziziphus mauritiana Lamk.).

Recently, SVM, an artificial intelligence technique has been developed as a support vector classification and support vector regression. The support vector machines based on the superior structural risk minimization principle offer a lot of advantages over the traditional techniques ANN and MLR such as unique, global and optimal solution. The most commonly used MLR technique suffers from multiple local solutions, over-fit to data and often leads to poor generalizability. This means good performance for training dataset and poor performance for unseen test dataset [21]. Thus, in the present work, the issue has been address to explore the use of support vector regression to predict and analyze the mass of ber fruits. This may be effective tools for enhancing the fruit grading system. However, emerging relationships between output and input parameters using ANN and SVM approaches do not require a priori knowledge of the model building process [22, 23]. In agricultural applications, in particular, ANN models have been designed for sorting, grading, identification, and prediction [7, 24, 25]. In addition, SVM technology in such agricultural applications has been explored in several reports. Vapnik [26] proposed the SVM method, which is a promising methodology for modeling data. The SVM is a learning method that uses linear tasks in a high-dimensional feature space and is trained with a training procedure from optimization theory [22]. Unlike the ANN model, which attempts to decrease the error on the learning data, SVM seeks to minimize an upper bound of the generalization error based on the structural risk minimization principle [22, 27].

The objectives of the present study are to build multiple linear regression (MLR), ANN, and support vector regression (SVR) models to predict ber fruit mass based on axial dimensions, to compare the performance of the established models in term of precision and accuracy given their obtained prediction values. The findings of this study can help improve a fruit grading system based on the prediction of the mass of ber fruits.

## Materials and methods

### Ber fruit samples

**Fig 1:**
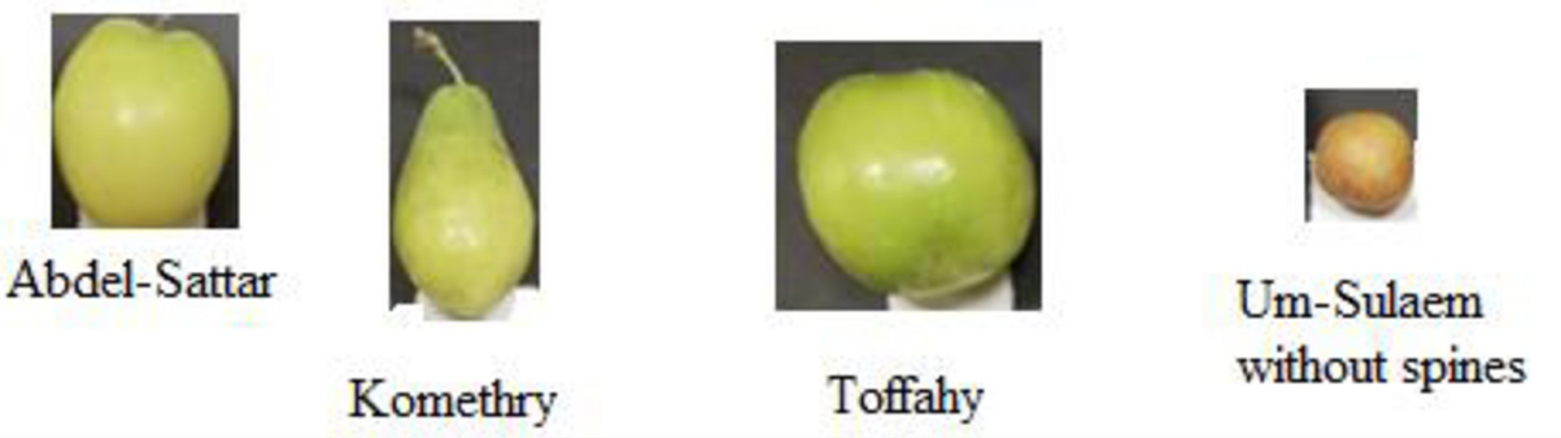
Investigated ber cultivars.

The present study was conducted in the 2020 season on 12 years old of four ber *Ziziphus mauritiana* Lamk. cultivars (Fig 1), namely, Komethry (V1), Toffahy (V2), Um-Sulaem without spines (V3), and Abdel-Sattar (V4), budded on ber rootstock in the orchard of the Research and Agricultural Experimental Station at Dirab region, King Saud University, Riyadh, Saudi Arabia (GPS coordinates: 24^o^24'43.0"N latitude, 46^o^39'30.7"E). The trees were planted in sandy soil at a spacing of 4 × 5 meters and irrigated by a drip irrigation system. In addition, they received traditional agricultural treatment, which is usually applied in this orchard.

For the present study, 12 trees were selected that were as uniform as possible in growth and health, and with three replicates for each cultivar and a single tree for each replicate (i.e., 4 cultivars × 3 replicates = 12 trees). The cultivars were arranged in a randomized manner. Samples of 50 mature fruits were taken from each tree at random during the end of winter when the fruit color turned light green (ovary green), and were cleaned manually to remove all foreign material. Thus, a total of 600 patterns were obtained.

**Fig 2:**
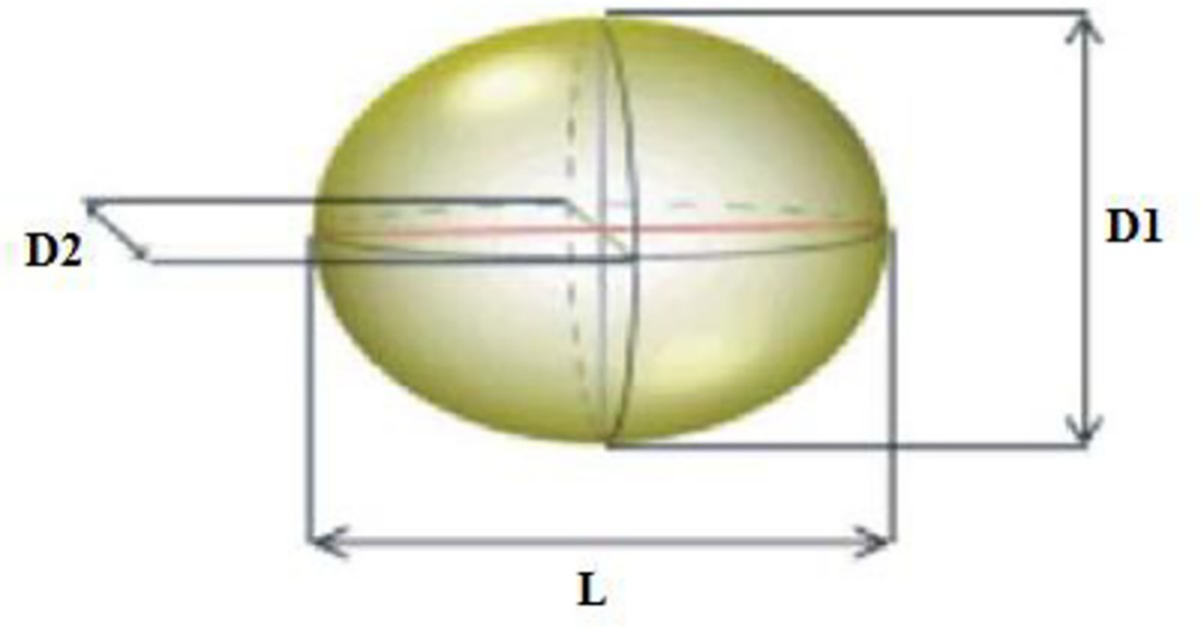
Three perpendicular axes: Length (L), major diameter (D1), and minor diameter (D2) of a ber.

The axial dimensions and mass of the randomly selected fruits from each cultivar were determined. The mass of each fruit (*M*) was measured by employing a digital balance (Metler Toledo; ±0.001 g). Three perpendicular axial dimensions–length (L), major diameter (D1), and minor diameter (D2)–for single fruits were recorded using a digital vernier caliper (Mitutoyo, Japan, ±0.01 mm), as illustrated in Fig 2. These measurements were performed at the Fruit Laboratory located at the College of Food and Agriculture Sciences, King Saud University, Riyadh, Saudi Arabia. For modeling purposes, the cultivars were marked with 0 and 1, as illustrated in the sample data in Table 1. For the data used in the modeling process, the descriptive statistics are presented in Table 2 to simplify the model. L, D1, and D2 were used as input variables while the ber mass was considered the model output for four cultivars.

**Table 1:** Ber cultivars ware marked with 0 and 1 for modeling purposes.

Komethry	Toffahy	Um-Sulaem without spines	Abdel-Sattar	L	D2	D1	M
0	1	0	0	40.99	43.91	43.45	43.15
0	0	1	0	20.7	24.75	25.38	7.06
0	0	0	1	35.41	31.26	33.49	18.45
1	0	0	0	44.81	26.07	27.04	16.86

**Table 2:** Minimum (Min), maximum (Max) and standard deviation (Sd) for the data used in the modeling process.

	Training dataset (n = 480)				Validation dataset (n = 120)			
	Min	Max	Mean	Sd	Min	Max	Mean	Sd
Model input								
L (mm)	20.1	47.88	35.26	9.21	19.89	47.19	36.58	7.91
D2 (mm)	23.38	44.17	30.94	7.12	24.12	44.39	32.99	7.25
D1 (mm)	24.79	44.65	31.95	7.24	25.01	44.44	33.93	7.11
Model output								
Ber fruit mass (g)	4.91	41.12	19.65	11.66	7.06	43.15	23.08	11.88

### Algorithms applied in ber fruit mass modeling

**Fig 3:**
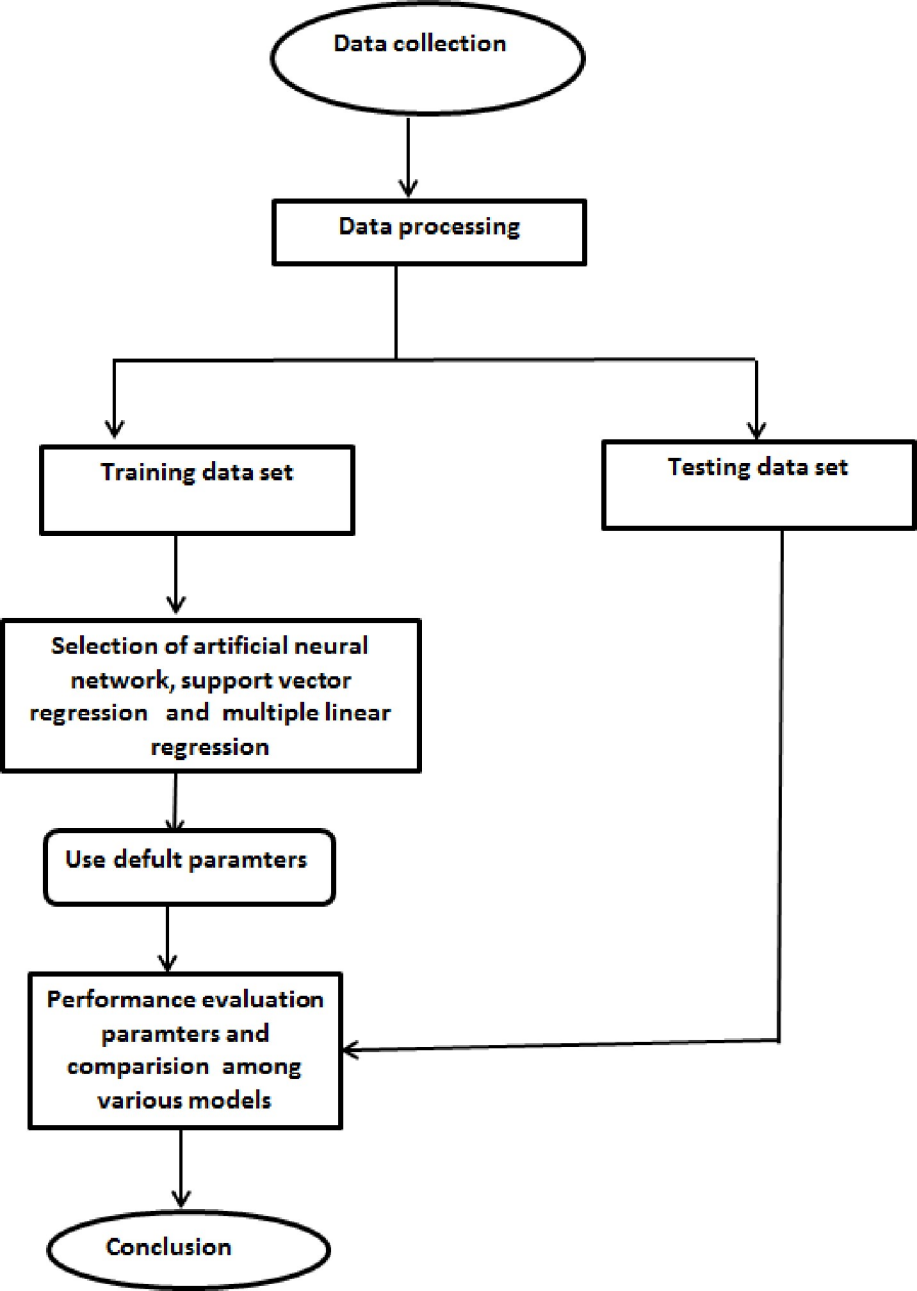
A flowchart for the current study for algorithms applied in ber fruit mass modeling.

The collected dataset corresponding to 600 patterns was directed by Weka software to select training and test datasets with 480 patterns (80%) and 120 patterns (20%) randomly, respectively. The training dataset was used to create the MLR, ANN, and SVR models, while the test dataset was used as unknown data to determine the generalizability of the established models. A flowchart for the current study for algorithms applied in ber fruit mass modeling is shown in Fig 3.

### Multiple linear regression

Regression is a modeling task that involves predicting a numeric value given an input. Linear regression is the standard algorithm for regression that assumes a linear relationship between inputs and the target variable. An MLR model has a general form as follows:

$${\hat{y}}_{i}=\beta_{0}+\sum_{i=1}^{n}\beta_{i}X_{i}+e_{i},i=1,2,\ldots n,$$ (1)

Where ${\hat{y}}_{i}$ is the mass in the *i*^th^ sample, *X*_*i*_ corresponds to input parameters (V1, V2, V3, V4, L, D1, and D2), β_*0*_ is the equation intercept, β_*i*_ is the linear regression coefficient for inputs, and *e*_*i*_ is the error. Data linked to the training dataset were fitted to (1) by means of the Waikato Environment for Knowledge Analysis (Weka) software, which is an open source data mining tool. The simplest mode for using Weka is through a graphical user interface called Explorer. This provides access to all of its facilities using menu selection and form filling. Moreover, the Weka workbench is known as easy-to-use and robust software for data mining [28].

### ANN model

An ANN is an approach modified from biological neural networks. In particular, it has the capacity to predict the system targets in exchange for exact input variables with biases and weights as a learning procedure. The significant information that reaches ANN nodes is determined by adjusted weights and biases. Furthermore, at a later stage of training the ANN model, the adjusted biases are values that are added to weights. The ANN training process is dependent on decreasing the error that results from the differences between the observed and predicted outputs. Because it is a training procedure, it repeatedly attempts to adjust and enhance the biases and weights to suggest the best target. It is worth noting that the function estimate is considered one of the greatest vital applications of ANN models.

**Fig 4:**
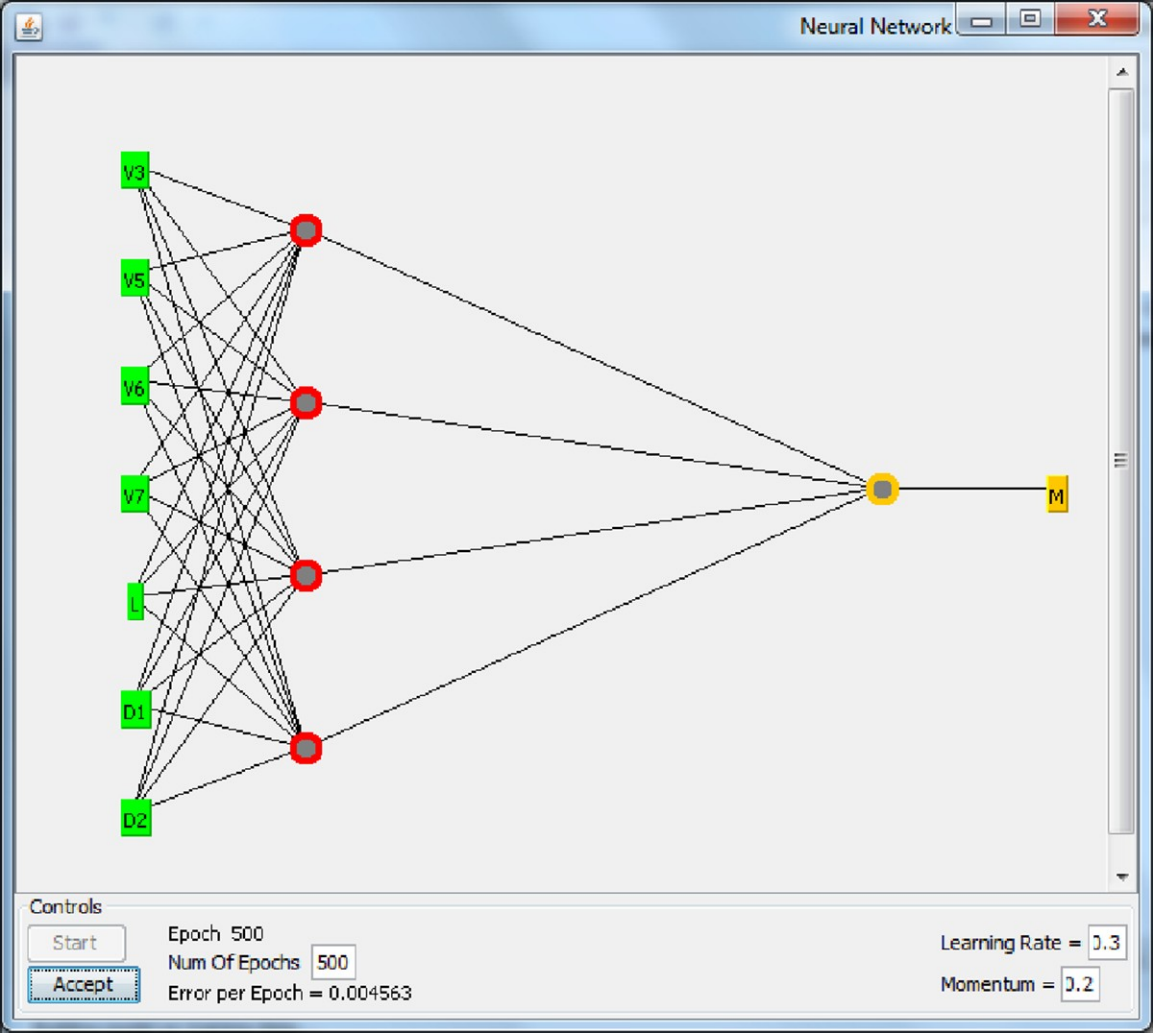
ANN created by Weka for ber fruit mass modeling.

Weka tool was configured to perform the training of a multilayer perceptron for background on multilayer perceptrons and neural networks. All of the nodes in the multilayer perceptron use a standard sigmoid function [29]. In this study, a feed-forward three-layer ANN model trained by the back-propagation method was generated using Weka software with default values. The sigmoid transfer function, the only activation function in Weka, was used for the output and hidden layers. Seven input parameters corresponding to V1, V2, V3, V4, L, D1, and D2 were used as neurons in the input layer of the ANN model. Fig 4 presents a schematic representation of the network created by Weka software. A three-layer feed-forward ANN model was the default type in Weka software. A total of 480 data patterns (training dataset) were employed to train the established ANN model. A normalization process within the range [–1, 1] for input and output values was achieved by the Weka tool prior to the training process. This process aims to complete the training operation easily and to ensure that the output data fall into the exact range [30]. Fig 4 shows number of layers (3), number of neurons at the input layer (7 neurons), hidden layer (1 with 4 neurons) and output layer (1 neuron). The momentum, learning rate, training epoch and training error are appeared on the Fig 4 to be 0.2, 0.3, 500 and 0.004563, respectively.

### Support vector regression

**Fig 5:**
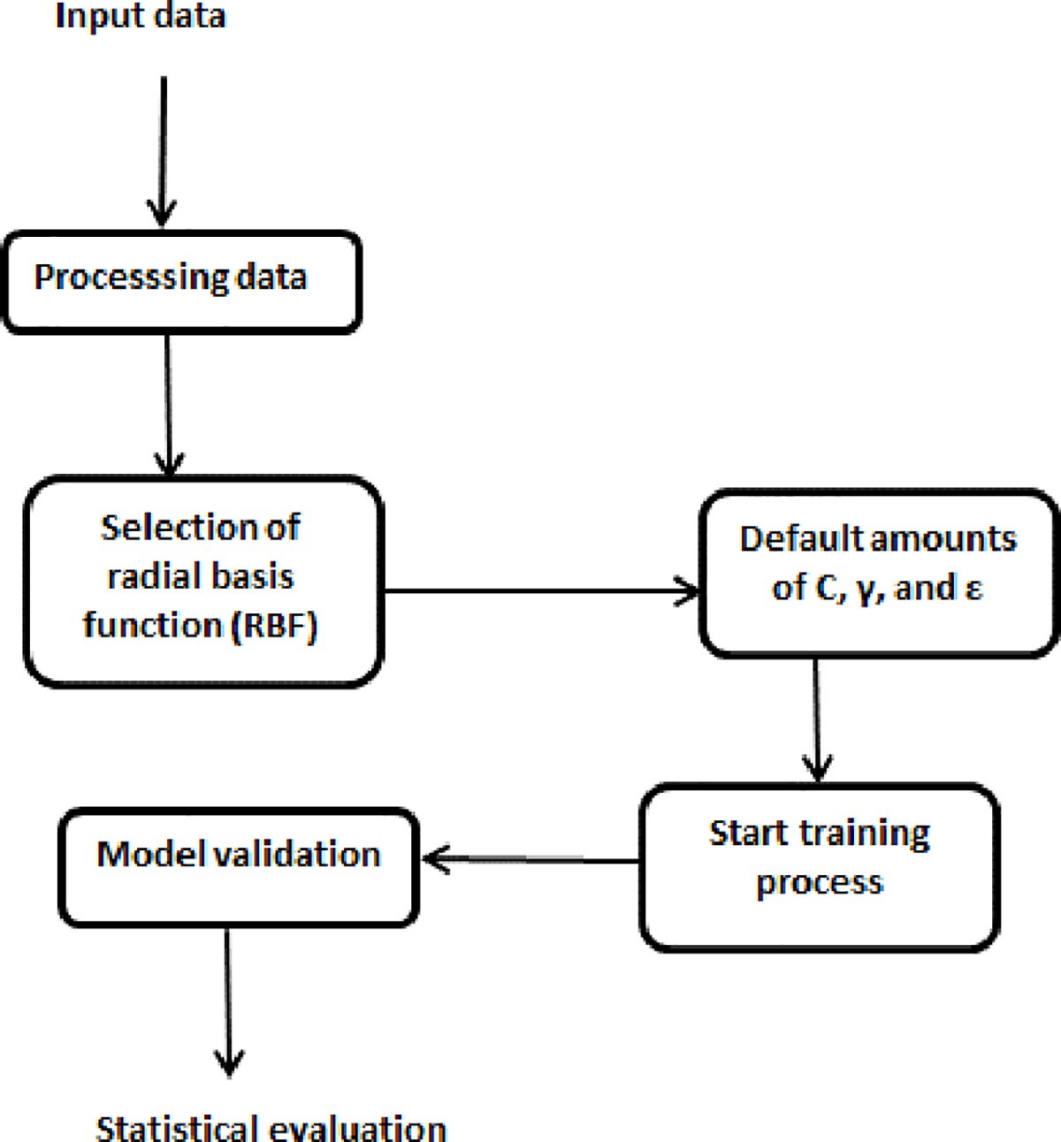
Schematic configuration of the SVR model using Weka (adapted from [38]).

For regression and classification applications, Vapnik [31] developed and introduced the SVM, which is considered as a function approximation. It is a promising, computationally powerful technique when there are no prior assumptions for modeling purposes. Generally, regression-based SVM is called SVR. SVR is created based on the principle of the structural risk minimization to solve complex problems. SVR finds the linear regression task, given by (2):

$$F\left(x\right)=w^{T}x+b,$$ (2)

where *b* and *w* denote the regression coefficients. According to Cristianini and Shawe-Taylor [22] and Gunn [32], the optimal regression model (2) can be determined by

$$\mathrm{Minimize}\frac{1}{2}{\left\|w\right\|}^{2}+C\sum_{i=1}^{N}\left(\zeta_{i}+\zeta_{i}^{*}\right)\;Subject\;to\left\{ \begin{array}{l} \;F\left(x\right)-y_{i}\leq \varepsilon +\zeta_{i}^{*}\\ \;y_{i}-F\left(x\right)\leq \varepsilon +\zeta_{i}\\ \zeta_{i},\zeta_{i}^{*}\geq 0,\;i=1,2,\ldots ,N \end{array}\right.$$ (3)

where *ε* is the *ε*- insensitive loss error function, C is the regularization factor (also called penalty parameter), C > 0, and *ζ_i_* and $\zeta_{i}^{*}$ are slack variables that denote upper and lower limitations on the regression model. Hereafter, to minimize (2) subject to (3), the function is given by Gunn [32] and Cimen [33]:

$$f\left(x\right)=\sum_{i=1}^{N}\left(\alpha_{i}^{*}-\alpha_{i}\right)K\left(x,x_{i}\right)+B,$$ (4)

where the function *K(x*, *x*_*i*_*)* is called the kernel function, *B* is the bias value, and $\alpha_{i}^{*},\alpha_{i}$ ≥ 0 are Lagrange multipliers. In the literature, different forms of kernel functions are adapted to solve a problem by SVR [34]. In the present study, a kernel function called the radial basis function (RBF) is employed for creating the SVR model, as it is widely used in agricultural applications [35, 36]. The advantages of the selected RBF are its simplicity, fast convergence, and satisfactory performance in high-dimensional spaces [37]. The equation of the RBF kernel function is as follows:

$$k\left(x,x_{i}\right)=exp\left(-\gamma {\left\|x-x_{i}\right\|}^{2}\right),$$ (5)

where C, γ, and ε are three predefined parameters whose values were set to 1, 0.01, and 0.001, respectively, which are the default quantities used in the Weka software. However, these parameters are usually selected and used as a black box, without understanding the internal details. Also, other kernel functions like NormalizedPolyKernel, Puk and PolyKernel are tested with the default quantities used in the Weka software. Fig 5 presents the procedure for building the SVR model in the Weka software.

### Evaluation parameters

In the present study, to examine the accuracy of the investigated models for ber fruit mass prediction, different statistical evaluation parameters were employed. The root mean squared error (RMSE), which measured the difference between the predicted and actual ber fruit mass based on the average error, can be calculated as follows:

$$RMSE=\sqrt{\frac{1}{n}\sum_{t=1}^{n}{\left(Y_{t}-F_{t}\right)}^{2}},$$ (6)

where Y_t_ and F_t_ are the actual value and predicted value, respectively, and the number of observations is denoted *n* in the testing and training datasets. The mean absolute error (MAE) was also used to measure the closeness of the actual ber fruit mass to the predicted mass. The precise prediction of the ber mass is defined by lower value of MAE and is calculated as follows:

$$MAE=\frac{1}{n}\sum_{t=1}^{n}\left|Y_{t}-F_{t}\right|$$ (7)

The degree of linearity between the predicted and actual values of ber fruit mass was also determined by calculating the correlation coefficient R. If Y_t_ is the actual value and F_t_ is the predicted value, the correlation coefficient is calculated as follows [39]:

$$R=\frac{\sum_{t=1}^{n}\left(Y_{t}-\bar{Y}\right)\times \left(F_{t}-\bar{F}\right)}{\sqrt{{\sum_{t=1}^{n}\left(Y_{t}-\bar{Y}\right)}^{2}\times }\sqrt{\sum_{t=1}^{n}{\left(F_{t}-\bar{F}\right)}^{2}}}$$ (8)

The coefficient of correlation takes values from –1 to +1. A correlation coefficient with a positive value implies that the two variables fluctuate in the same direction with respect to their means. A correlation coefficient with a negative value suggests that the two variables vary in reverse directions with respect to their means. A value close to 0 implies that the two variables have a small linear relationship. Data analysis and prediction of the adequacy of model were performed using an Excel spreadsheet.

## Results

**Fig 6:**
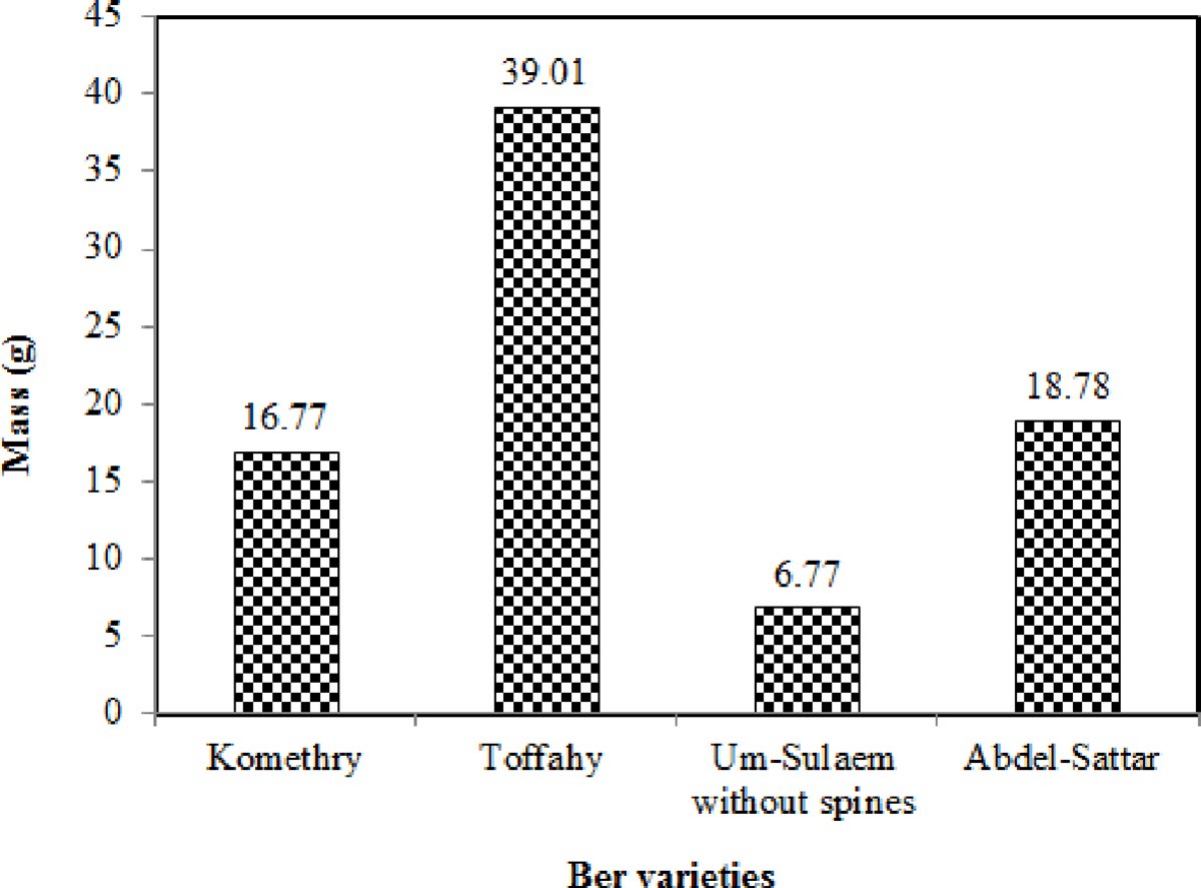
Mass of ber fruits for the investigated cultivars.

Fig 6 depicts the values of the mass of ber fruits for the studied cultivars. It is evident that the Toffahy cultivar had the largest mass compared to other cultivars, with a mean of 39.01 g. The Um-Sulaem without spines cultivar had the lowest mass with a mean of 6.77 g, while the remaining cultivars (Komethry and Abdel-Sattar) had a mean mass of 16.77g and 18.78 g, respectively, as displayed in Fig 6.

**Fig 7:**
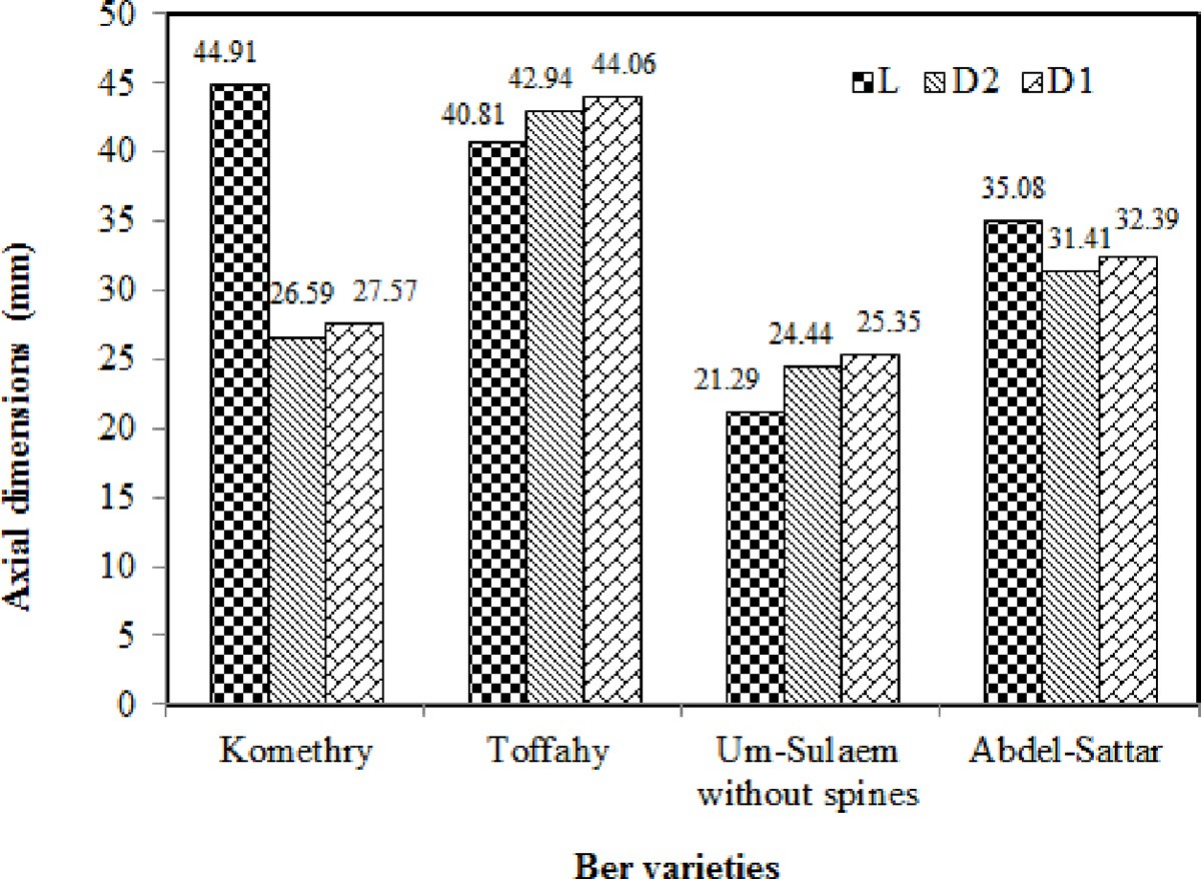
Distribution of the mean axial dimension values of ber fruits for the investigated cultivars.

Fig 7 depicts the distribution of the average axial dimension values of ber fruits for the studied cultivars. It can be seen that the Komethry cultivar had the longest length with a mean of 44.91 mm, while the Um-Sulaem without spines cultivar had the lowest length with a mean of 21.29 mm. It should be noted that the D1 dimension was always larger than the D2 dimension for all cultivars. The Toffahy cultivar had the largest D1 and D2 dimensions with a mean of 44.06 mm and 42.94 mm, respectively, for D1 and D2. The Um-Sulaem without spines cultivar had the lowest D1 and D2 with a mean of 25.35 mm and 24.44 mm, respectively. However, in the study by Al-Obeed [40], the fruit length for the Komethry, Um-Sulaem, and Toffahy cultivars in the 2005 season was recorded as 5.83, 3.31, and 4.04 cm, respectively, while they were 5.87, 3.19, and 3.96, respectively, in the 2006 season. In addition, in the study by Al-Obeed [40], the fruit diameter for the Komethry, Um-Sulaem, and Toffahy cultivars in the 2005 season was recorded as 2.72, 2.90, and 4.00 cm, respectively, while they were 2.87, 2.92, and 4.01 cm, respectively, in the 2006 season.

The first task in analysis of regression was to predict the coefficients appearing in (1). These coefficients were captured using the Weka tool using the training dataset. The adequacy of the MLR model was expressed by the *R*^2^ value, which was established to be 0.595 and 0.4614 (Table 3) for the training and validation datasets, respectively, indicating that 59.5% of the variability in the ber fruit mass could be explained by the established regression model when it encountered new data. The lower R^2^ may be due to the non-linear relationship between L, D1, and D2 and mass. Khoshnam et al. [41] used the physical characteristics of the pomegranate fruit to model the mass, and revealed that pomegranate mass was determined based on the minor diameter as a nonlinear relation. Additionally, Ebrahimi et al. [42] found that the minor diameter of the walnut had a nonlinear relation with its mass. The MLR model for ber mass estimation is as follows:

$$\begin{array}{l} M=-0.5618-1.8882*V1-7.8187*V2-8.3072*V3-1.6233*V4+0.1041*L\\ \;+0.2426*D2+0.2979*D1 \end{array}$$ (9)

**Fig 8:**
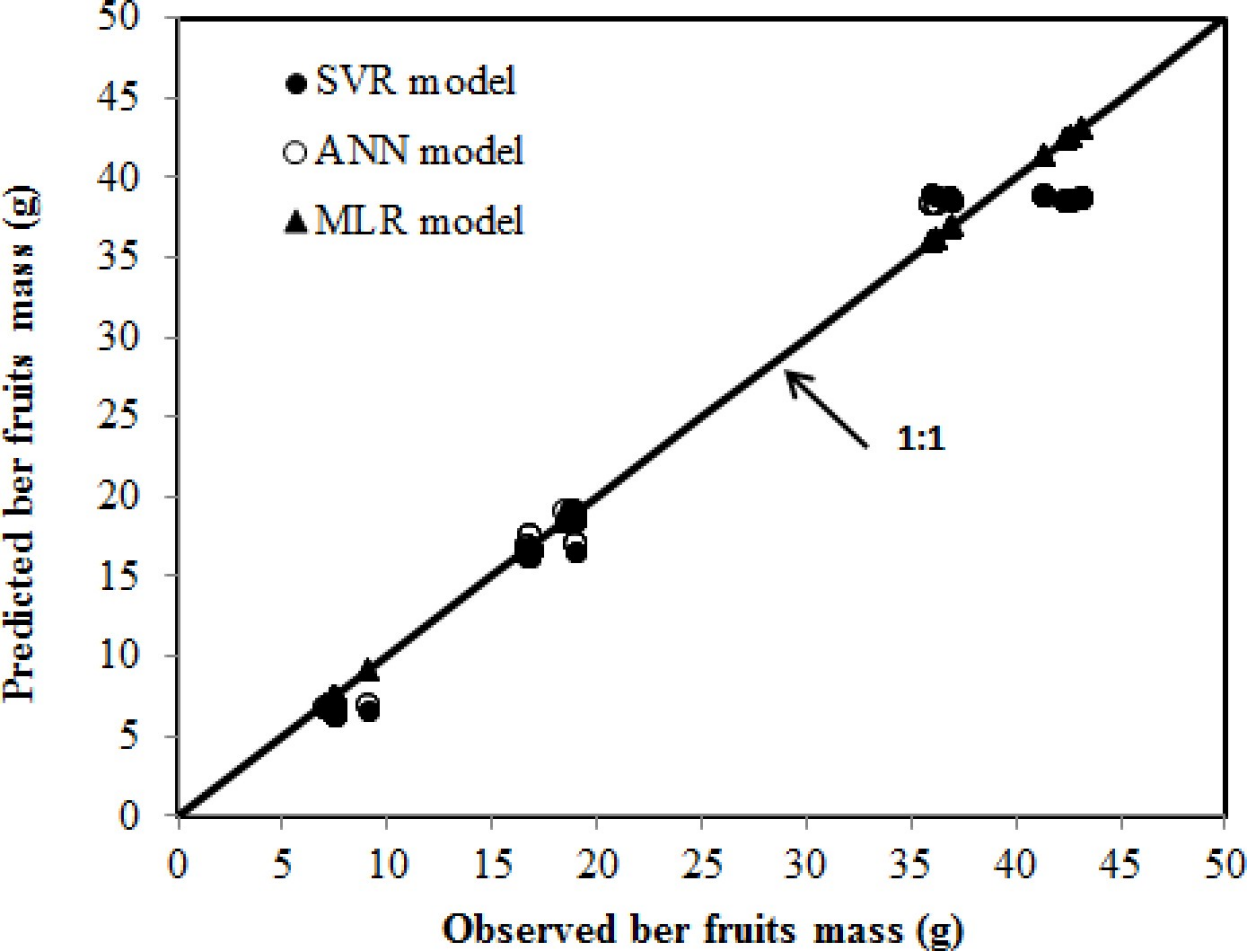
Relationship between observed and predicted ber fruit mass using the SVR, ANN and MLR models in the validation stage.

The statistical criteria derived from the MLR, SVR, and ANN models to predict the ber fruit mass are displayed in Table 3. The plots of observed ber fruit mass in the validation dataset against the predicted values are presented in Fig 8 using all models. The data distribution in the plot (Fig 8) indicates good agreement between the predicted and actual values of ber fruit mass for the datasets fed to the developed SVR and ANN models. However, average error [(predicted- actual)/n] values as depicted in Table 3 are -6.4230 g, -0.5294g and -0.5828g for MLR, ANN and SVR, models, respectively.

**Table 3:** Calculated goodness-of-fit values on support vector regression (SVR), multiple linear regression (MLR), and artificial neural network (ANN) models of ber fruit mass estimation.

Statistical criteria	Validation			Training		
	MLR	ANN	SVR	MLR	ANN	SVR
R^2^	0.4614	0.9780	0.9771	0.5950	0.9947	0.9947
RMSE (g)	11.3742	1.8479	1.8814	9.4591	0.8790	0.9714
MAE (g)	2.5636	1.1279	1.1380	4.4059	0.7515	0.8042
Average error (g)	-6.4230	-0.5294	-0.5828	-4.4778	0.2334	0.0044

Table 4 depicts, performance of the different kernel functions of SVR in the validation dataset with the default quantities used in the Weka software. As shown in Table 4, it is noticeable that all the tested kernel functions provided good performance with R^2^ over 0.9 where the best result was achieved with RBF Kernel with R^2^ of 0.9771 and less MSE and RMSE compared with other kernel functions. This behavior may be attributed to the data have a nonlinear relationship between the features [43].

**Table 4:** Performance of the different kernel functions of SVR in the validation dataset with the default quantities used in the Weka software.

Kernel functions	Scheme	R^2^	MAE	RMSE
NormalizedPolyKernel	Scheme:weka.classifiers.functions.SMOreg -C 1.0 -N 0 -I "weka.classifiers.functions.supportVector.RegSMOImproved -L 0.001 -W 1 -P 1.0E-12 -T 0.001 -V" -K "weka.classifiers.functions.supportVector.NormalizedPolyKernel -C 250007 -E 2.0"	0.9765	1.2953	1.905
PolyKernel	Scheme:weka.classifiers.functions.SMOreg -C 1.0 -N 0 -I "weka.classifiers.functions.supportVector.RegSMOImproved -L 0.001 -W 1 -P 1.0E-12 -T 0.001 -V" -K "weka.classifiers.functions.supportVector.PolyKernel -C 250007 -E 1.0"	0.9765	1.2688	1.8869
Puk	Scheme:weka.classifiers.functions.SMOreg -C 1.0 -N 0 -I "weka.classifiers.functions.supportVector.RegSMOImproved -L 0.001 -W 1 -P 1.0E-12 -T 0.001 -V" -K "weka.classifiers.functions.supportVector.Puk -C 250007 -O 1.0 -S 1.0"	0.9761	1.278	1.9375
RBFKernel	Scheme:weka.classifiers.functions.SMOreg -C 1.0 -N 0 -I "weka.classifiers.functions.supportVector.RegSMOImproved -L 0.001 -W 1 -P 1.0E-12 -T 0.001 -V" -K "weka.classifiers.functions.supportVector.RBFKernel -C 250007 -G 0.01"	0.9771	1.1380	1.8814

ANN offers a more advanced means of prediction than traditional statistical methods, as they make it possible to provide inputs in a computer or calculating device and evaluate the expected fruit weight [44]. The plots demonstrate that the curves generated by the ANN and SVR models and the curves formed by the actual observed values have the same pattern and shape, which demonstrates that the ANN and SVR successfully modeled the data using a dependable structure with seven nodes. The results demonstrate that by using the ANN and SVR models, specialists in the grading system field can place data in the developed spreadsheet and predict the cost-effectiveness of the ber fruit mass in advance.

## Discussion

In various agricultural applications, the ultimate goal of SVR and ANN is to build models that provide more accurate and precise prediction values of the target variables. The performance of the SVR and ANN models can be evaluated by performing a comparison calculation between actual and predicted desired values based on the examined inputs. Both SVR and ANN models were able to precisely predict the ber fruit mass of four different cultivars with R^2^ = 0.978 and 0.977 (Table 3), respectively, for the test dataset that was not used in the learning phase. Additionally, the trained ber fruit mass models had relatively stable statistical criteria, suggesting that for the test and training datasets, overlearning did not occur during the training phase (Table 3), and the established models had satisfactory generalizability when handling an entirely new dataset [45, 46].

To assess the performance of models, the R^2^ value is commonly employed to evaluate the prediction accuracy of a definite model, while the MAE and RMSE are generally used to display the precision of a model based on residual analysis. Therefore, it is desired to use a combination of measures to determine and/or compare the overall performance of models. The statistical criteria values in modeling ber fruit mass based on the ber cultivar type and three axial dimensions were represented by R^2^, MAE, and RMSE corresponding to the SVR, MLR, and ANN models. These values indicated a fairly high precision and higher accuracy of prediction for SVR and ANN models for both the validation and training datasets (Table 3) in comparison with the MLR model. The magnitude of the average error value for the investigated models reveals over/underprediction concerning the mean of the observed average error values (Table 3). In addition, the SVR and ANN models have small average error values between the predicted and actual ber fruit mass.

The superior performance of SVR and ANN models over the MLR model was mostly due to several required assumptions, such as the linear function being regressed when the traditional MLR was applied [38]. Thus, the application of the established MLR model may be particularly limited. In contrast, in recognizing patterns and fitting different functions in different type of data, the SVR and ANN models had good performance. However, according to Ahmadi and Rodehutscord [38], to create an effective prediction model, SVR and ANN approaches require more data samples than the traditional MLR model. SVR and ANN models may also behave well when an adequate dataset is accessible, and they are statistically well scattered in the input and output space.

The main advantages of ANN and SVR over predictable regression are as follows: (1) There is no prior information when ANN and SVR models are applied to fit a function, and (2) SVR and ANN have a general approximation ability and can estimate nearly all types of non-linear relationships, whereas the MLR model is only effective for linear relationships [30, 38, 46, 47].

However, there are some restrictions for both the SVR and ANN modeling methods. In these procedures, standardized coefficients matching each parameter may not be easily determined as they are in ordinary regression models. SVR and ANN procedures, which perform regression analyses, yield weights that are difficult to understand, as they are typically influenced by the computer software used to produce them [38]. Thus, they in fact use a black box method, which does not offer comprehensive insight into the internal mechanisms of the model or information for appraising the interaction of inputs [23]. Furthermore, there may be difficulty sharing the established SVR and ANN models with other researchers. In contrast, the generated MLR model can be applied after knowing the regression coefficients to achieve simple calculations to predict an output (e.g., ber fruit mass or others). To share established SVR and ANN models, it is necessary to deliver the production code of the trained SVR model or the values of the adjusted bias and weight matrices of ANN model, which may be very complex and large, to a specific program or computer software to create an interactive computer tool for predicting the mass of a new ber fruit.

## Conclusion

Grading fruits based on mass is important in packaging and reduces the waste, also increases the marketing value of agricultural produce. The current study proposed SVR and ANN approaches to predict ber fruit mass of four ber fruits cultivars. The established SVR and ANN models yielded better prediction values for ber fruit mass estimation (using cultivar type and three axial dimensions) than those yielded by traditional MLR. In addition, there were no noticeable differences between the performance of the SVR and ANN models. The results suggest that the ANN method may be capable of increasing the ability to accurately design a grading system of ber fruit cultivars.

## Supporting information

S1 Data (XLSX)
